# Analysis of Student Engagement With AI in Undergraduate Lectures: Protocol for an Implementation Study

**DOI:** 10.2196/92990

**Published:** 2026-10-21

**Authors:** Mthobisi Luca Ntuli, Nomakhosi Mpofana, Michael Paulse, Godfrey Nkululeko Mazibuko, Shamanie Govender

**Affiliations:** 1 Mathematics Department, Faculty of Applied Sciences, Durban University of Technology, 121 Steve Biko Road, Musgrave, Berea, Durban, KwaZulu-Natal, 4001, South Africa, 27 787811946; 2 Department of Somatology, Health Sciences, Durban University of Technology, Durban, KwaZulu-Natal, South Africa; 3 Graduate School of Business, University of Cape Town, Cape Town, Western Cape, South Africa; 4 Department of Biomedical and Clinical Technology, Faculty of Health Sciences, Durban University of Technology, Durban, KwaZulu-Natal, South Africa

**Keywords:** artificial intelligence, AI, AI literacy, undergraduate learning, applied sciences, health sciences, mixed methods

## Abstract

**Background:**

AI has quickly become integrated into higher education, transforming how students access information, complete academic tasks, and engage with learning. Universities of technology, with their focus on applied learning, technological skills, and graduate employability, face both challenges and opportunities in incorporating AI in ways that improve learning without sacrificing conceptual understanding, critical thinking, or academic integrity. While current research highlights widespread student use of AI tools, there remains limited empirical evidence on how undergraduate students in applied and health-related fields interact with AI, perceive its educational impact, and manage risks associated with overreliance.

**Objective:**

The study aims to examine undergraduate students’ AI use patterns, their perceptions of how AI affects conceptual understanding and critical thinking, and their views on risks such as dependency and ethical issues within a university of technology setting. An additional goal is to investigate students’ experiences with AI literacy programs and gather evidence to support responsible, educationally grounded AI integration that aligns with institutional strategies and graduate attributes.

**Methods:**

The study adopts a convergent mixed methods, cross-sectional, exploratory-descriptive design. Quantitative data will be collected using a structured questionnaire administered to undergraduate students across the mathematics, somatology, and Bachelor of Health Sciences (biomedical and clinical technology) programs within the Faculties of Applied Sciences and Health Sciences at Durban University of Technology. Qualitative data will be generated through semistructured interviews. Quantitative data will be analyzed using descriptive and inferential statistical methods, whereas qualitative data will be analyzed thematically using the method by Tesch. Integration of findings will occur at the interpretation stage to enable triangulation and comprehensive insight. Integration will follow the joint display approach described by Guetterman et al. Quantitative and qualitative results for each objective will be displayed side by side in matrices that compare statistical patterns with thematic insights. Convergence, complementarity, or discordance will be explicitly noted. Meta-inferences will be generated through team deliberation using an integration codebook. A triangulation protocol will document how discrepancies are resolved (eg, prioritizing qualitative depth for mechanism explanation and quantitative breadth for prevalence estimation).

**Results:**

A pilot study conducted within the mathematics department identified emerging patterns of AI dependence among students, helping refine research tools and focus interventions. This study was funded by the Department of Higher Education and Training on January 1, 2025, and was conducted between August 2025 and November 2025, with preliminary results available at the end of that period. Data collection is ongoing and is expected to conclude by the end of November 2026, after which the data will be analyzed in 2027 and the findings will be published in a separate results paper.

**Conclusions:**

The study is expected to provide evidence on the use of AI in applied and health-related fields. The findings will inform institutional policy, curriculum development, and the development of a short course on the responsible use of AI, thereby supporting broader discussions on the sustainable integration of AI in higher education.

## Introduction

### Background

The increasing use of AI tools in undergraduate lectures offers both opportunities and challenges [1]. While AI platforms can enhance learning by providing explanations, solutions, and feedback, there is growing concern that students may become overly dependent on these tools. This overreliance could bypass essential cognitive processes, potentially hindering the development of independent problem-solving skills and deep understanding [2,3].

The integration of AI in teaching and learning has gained significant attention in recent years, with scholars highlighting both its transformative potential and its associated risks. AI tools are increasingly used by students to solve mathematical problems; by skin rejuvenation therapists to enhance outcomes; and by clinical technology students to diagnose, treat, and provide patient support and care to improve quality of life, offering personalized feedback and enhancing engagement [4,5]. These tools can support conceptual understanding and foster creative problem-solving when used reflectively [5].

However, concerns have been raised about students’ overreliance on AI, often described as cognitive offloading, which may lead to superficial learning and hinder the development of independent reasoning skills [6,7]. When AI transitions from a tool for reflective practice and deep exploration to becoming a simple answer generator, students bypass the critical cognitive struggle and effort necessary for deeper learning and the solidification of procedural competence [8]. This dependence can diminish intrinsic understanding of complex methodological processes, creating a “black box effect” in which students prioritize obtaining the correct output generated by AI over understanding the underlying methods, leading to superficial learning in which answers are accepted rather than reasoned [9]. This aligns with the study’s research problem, which focuses on understanding how AI engagement affects students’ thinking and critical evaluation.

Theoretical frameworks such as constructivism and critical pedagogy provide a lens for interpreting student-AI interactions. Constructivism emphasizes active knowledge construction [10,11], whereas critical pedagogy encourages learners to interrogate AI outputs and their underlying assumptions [12]. A recurring theme in the literature is the lack of AI literacy among students, which limits their ability to use these tools effectively and critically [13,14]. While the existing literature praises AI’s potential as a scaffold, there remains a critical gap in empirical research concerning the specific tipping point at which AI transitions from an effective cognitive aid to a destructive substitute for genuine intellectual effort. Specifically, there is a paucity of longitudinal data that rigorously assess the relationship between habitual use of AI and a student’s long-term acquisition of transferable problem-solving skills and meta-cognitive awareness across varied academic disciplines. This current lack of evidence creates a perilous vacuum in educational policy and curriculum design, making it impossible to define effective guidelines for educational use or ensure that AI integration strengthens rather than undermines the foundational goals of education: critical thinking, skill mastery, and intellectual autonomy. This supports the study’s objective to develop pedagogical interventions that promote responsible AI use in mathematics education.

### Theoretical and Conceptual Underpinnings

This study draws on established theories of health sciences education and professional practice, as well as key concepts from the literature on the use of AI in undergraduate teaching and learning. Learning in health sciences is understood as an active, socially situated, and practice-oriented process grounded in constructivist and experiential learning perspectives that emphasize knowledge building through engagement, reflection, and the application of skills in real-world settings [15,16]. These views are especially relevant in undergraduate health sciences education, where students gain competence and develop their professional identity by participating in disciplinary practices and communities of practice [17].

Within this theoretical framework, AI-enabled educational tools are viewed as mediating resources that can support personalized learning, formative feedback, and self-regulated learning. The study is further guided by theories of technology-enhanced learning, particularly the technological pedagogical content knowledge framework, which emphasizes integrating technological, pedagogical, and disciplinary knowledge into effective teaching practices [13]. Additionally, concepts from self-regulated learning theory highlight how AI-supported feedback and adaptive learning systems can improve learners’ ability to plan, monitor, and reflect on their own learning [18]. Cognitive load theory also offers a perspective on how AI tools may assist learning by reducing unnecessary cognitive load and optimizing instructional design for complex health sciences content [19].

The study is also situated within broader outcome- and competency-oriented approaches that support modern health sciences education. Outcome-based education and competency-based approaches emphasize alignment among curriculum design, teaching strategies, assessment, and clearly defined graduate attributes [20,21]. These perspectives offer a useful framework for exploring how AI can support the development of knowledge, skills, and professional competencies in undergraduate health sciences programs.

Moreover, the study aligns with these theoretical perspectives by examining institutional and policy frameworks that shape curricula and teaching methods. Leading these are Durban University of Technology’s (DUT) Envision 2030 strategic goals [22] and relevant teaching policies and digital transformation guidelines [23,24]. These documents highlight innovative teaching methods, graduate flexibility, and the responsible use of digital technologies in higher education. Including these institutional priorities ensures that the study is relevant to the context and aligned with DUT’s strategic goals.

The conceptual framework emerging from these theories and policy positions integrates educational theory, AI-enabled pedagogy, and institutional context to guide the inquiry. While the framework is still being refined, it has informed the formulation of the research objectives and questions and will steer the study’s design, data collection, and interpretation of results.

### Aim

We aim to design, pilot, evaluate, and establish an effective approach for the ethical use of AI by students at a selected university of technology (UoT) in KwaZulu-Natal, South Africa. The goal is to create a framework for integrating AI into teaching and learning that improves education while preventing cognitive disengagement. This research contributes to the ongoing discussion of digital pedagogy and aims to inform curriculum development, teaching methods, and institutional policies related to AI in education.

### Objectives

The study will have the following objectives:

1. Investigate the patterns and purposes of AI use among undergraduate mathematics, somatology, medical aesthetics, and Bachelor of Health Sciences (BHSc) in biomedical and clinical technology students (first-fourth year)
2. Evaluate the impact on conceptual understanding and critical thinking
3. Identify the risks associated with overreliance on AI for students’ learning
4. Develop and assess pedagogical interventions such as AI literacy training that promote responsible and reflective use of AI tools

### Research Questions

The research questions are as follows:

1. How do undergraduate students across different disciplines and year levels use AI tools for academic purposes?
2. What is the impact of AI tool use on undergraduate students’ conceptual understanding and critical thinking skills?
3. What educational risks and dependencies are associated with student overreliance on AI tools?
4. How does an AI literacy intervention influence students’ adoption of responsible and ethical AI practices?

### Analytic Expectations

On the basis of cognitive offloading theory, we hypothesize that higher frequency of AI tool use will be negatively associated with self-reported critical thinking confidence (ρ<0; hypothesis 1), students who report verifying AI outputs will demonstrate higher AI literacy scores than nonverifiers (mean difference>0; Cohen *d*≥0.3; hypothesis 2), and postintervention AI literacy scores will show a statistically significant increase relative to baseline (2-tailed paired *t* test; *P*<.05; Cohen *d*≥0.4). These expectations guide inferential testing but remain exploratory given the study’s implementation focus.

### Context of the Study

The rapid integration of AI tools into higher education is transforming how students interact with course content. In undergraduate classrooms, students increasingly use platforms such as ChatGPT, Microsoft Copilot, and Wolfram Alpha to solve problems, verify answers, and grasp complex ideas. While these tools provide opportunities for personalized learning and instant feedback, they also pose risks, especially the potential weakening of independent problem-solving skills and critical thinking.

This study is part of the broader discussion on digital transformation in education and aligns with the theories of constructivism and critical pedagogy. Constructivism emphasizes active, student-centered learning where knowledge is constructed through interaction and experience. Critical pedagogy, on the other hand, encourages learners to question the tools and systems they use, including AI, to develop awareness of biases, limitations, and ethical issues.

The research addresses a critical educational concern: how to leverage the advantages of AI without hindering the development of deep conceptual understanding. It responds to observed trends in which some students passively accept AI-generated solutions, whereas others engage more critically and reflectively. The absence of formal AI literacy training further complicates this situation, emphasizing the need for structured interventions. By examining how students use AI in real-time classroom settings and assessing the effects of targeted teaching strategies, this study aims to design, pilot, evaluate, and establish an evidence-based framework for the ethical integration of AI into teaching and learning at a UoT in KwaZulu-Natal, South Africa, to enhance educational outcomes while promoting critical engagement and preventing cognitive disengagement among students. This research contributes to the ongoing discussion on digital pedagogy and aims to inform curriculum development, teaching methods, and institutional policies related to AI in education.

## Methods

### Overview

This study will use a convergent mixed methods research design in which qualitative and quantitative data are collected simultaneously, analyzed separately, and combined during interpretation to develop a comprehensive understanding of how undergraduate students use AI in learning. This approach is appropriate for exploring patterns of AI use, students’ perceptions of its impact on conceptual understanding and critical thinking, perceived risks of overreliance, and experiences with AI literacy interventions within applied and health-related disciplines at DUT.

Qualitative data will be collected through semistructured interviews, enabling detailed exploration of students’ experiences, meanings, and concerns regarding the use of AI. These data will be analyzed thematically using the method by Tesch [25], which enables systematic identification and interpretation of themes related to learning practices, critical engagement, risk, and ethical awareness.

Quantitative data will be collected using a structured questionnaire and analyzed using descriptive and inferential statistics to identify trends, associations, and pre- and postchange effects related to AI use and literacy (Multimedia Appendix 1). Combining qualitative and quantitative findings during interpretation will validate, complement, and clarify the results, thereby enhancing the study’s validity and explanatory power.

All statistical reporting will follow the JMIR Publications guidelines for reporting statistics, including effect sizes, CIs, and assumption checks for all inferential tests.

### Study Design and Setting

This study adopts a convergent mixed methods, exploratory-descriptive implementation design with a cross-sectional survey component and a pretest-posttest intervention phase. The cross-sectional component examines baseline AI use patterns, whereas the intervention component evaluates the impact of an AI literacy training module. The investigation is limited to undergraduate students’ use of AI in learning across the mathematics, somatology, and BHSc in biomedical and clinical technology programs. An exploratory-descriptive approach is appropriate as the study seeks to gain an in-depth understanding of students’ experiences, perceptions, and practices regarding the use of AI in an emerging, rapidly evolving educational context.

Data collection will be conducted in sequential and complementary phases, as outlined in the research design matrix (Table 1). Quantitative data will be collected through structured student surveys to identify patterns, frequencies, and purposes of AI use, as well as students’ self-reported perceptions of conceptual understanding, critical thinking, and potential risks associated with reliance on AI. These data will provide a broad overview of trends across disciplines and year levels.

**Table 1.** Research design matrix.

Objective (qualitative focus)	Qualitative research questions	Qualitative data and analysis	Quantitative variables	Statistical methods
Explore patterns and purposes of AI use among undergraduate students	How do students describe their use of AI tools across disciplines and year levels? For what academic purposes are AI tools used?	Focus groups Semistructured interviews Thematic analysis (method by Tesch [25])	Frequency of AI use Type of AI tool Academic purpose Discipline Year level	Frequencies and percentages Cross-tabulations Chi-square tests of association
Explore students’ perceptions of AI’s influence on conceptual understanding and critical thinking	How do students perceive the impact of AI on conceptual understanding? In what ways does AI support or hinder critical thinking?	Focus groups Interviews Thematic analysis (learning depth and reasoning)	Likert-scale perception scores Self-reported critical thinking confidence AI use frequency	Descriptive statistics (mean and SD) Cronbach α (scale reliability) Pearson or Spearman correlation
Explore perceived risks associated with overreliance on AI in learning	What risks do students associate with excessive AI use? How does AI dependence affect learning autonomy and integrity?	Focus groups Interviews Thematic analysis (risk, dependency, and ethics)	AI dependency indicators Risk awareness scores Verification behaviors	Frequencies and percentages Cross-tabulations Chi-square tests Spearman correlation
Explore experiences and perceived impact of AI literacy interventions	How do students experience AI literacy training? What changes occur in attitudes and practices following the intervention?	Reflective journals Focus groups Thematic analysis (reflection and responsibility)	AI literacy scores (pretest-posttest) Ethical awareness scores Responsible use indicators	Paired *t* test or Wilcoxon signed-rank test Effect size (Cohen *d*) Descriptive statistics

Qualitative data will be generated through focus group discussions and semistructured interviews to explore students’ experiences and perceptions in greater depth. This qualitative component will enable a nuanced understanding of how AI tools support or hinder learning, the nature of the risks associated with overreliance, and students’ reflections on the responsible use of AI. Where applicable, lecturer perspectives may be included to strengthen data triangulation.

The AI literacy intervention consists of a 4-week modular short course delivered through a blended learning approach, with weekly sessions combining facilitated workshops and complementary asynchronous online activities. Content covers (1) foundational AI concepts and tool evaluation, (2) ethical use and academic integrity, (3) critical appraisal of AI outputs, and (4) reflective integration strategies. Each session combines short lectures, interactive case studies, and guided practice with AI tools. Sessions are facilitated by Center for Excellence in Learning and Teaching instructional designers (responsible for curriculum design and fidelity monitoring) and subject lecturers (responsible for delivery and contextualization). Assessment is integrated across the 4 weeks and includes (1) weekly reflective journal entries to promote meta-cognitive awareness and critical engagement with AI use, (2) one group assignment focused on collaboratively evaluating and applying AI tools to discipline-specific teaching or learning scenarios, and (3) one individual assignment requiring a critical analysis and justified integration of AI into an academic task. Both the group and individual assignments are due in week 4, allowing students to consolidate learning across all modules. Formative feedback is provided throughout to support learning progression. Completion is tracked via learning management system (LMS) analytics, attendance registers, and short end-of-module quizzes, whereas fidelity is monitored through session checklists and facilitator debriefs.

Quantitative data will be analyzed using descriptive and inferential statistics, including frequencies, means, and comparisons. Qualitative data will be analyzed inductively using the method by Tesch [25] for thematic analysis to identify recurring patterns, themes, and meanings grounded in participants’ accounts. Integration of quantitative and qualitative findings will occur during the interpretation phase, enabling a comprehensive understanding of AI use, its educational implications, and the effectiveness of pedagogical interventions.

### Study Population

The target population for this study includes undergraduate students enrolled in the Faculty of Applied Sciences and the Faculty of Health Sciences at DUT. Participants will be selected from 3 academic departments: the Department of Mathematics, the Department of Biomedical and Clinical Technology and Biotechnology, and the Department of Somatology. Both male and female undergraduate students from these departments will be eligible to participate.

A purposive sampling strategy will recruit undergraduate students from years 1 to 4 in mathematics, somatology, and the BHSc in biomedical and clinical technology. Inclusion criteria are as follows: (1) current enrollment in the target programs, (2) age of 18 years or above, and (3) self-reported use of at least one AI tool for academic purposes in the previous semester. Exclusion criteria are as follows: (1) postgraduate students and (2) staff members. The target sample size is 384 participants, calculated using the Cochran formula for infinite populations at a 95% confidence level and a 5% margin of error, adjusted for design effect (1.3) and an anticipated 30% nonresponse rate. Recruitment will take place via departmental coordinators, LMS announcements, and in-class invitations. Response rate will be monitored weekly; if recruitment lags, reminder communications will be used. Students from selected departments will be invited to participate based on their availability and the relevance of their work to the study’s objectives, thereby enabling the collection of informed insights into their knowledge and use of AI in their respective fields.

To accommodate students with limited or no online access, trained independent research assistants will administer paper-based questionnaires. Responses from the hard copies will then be entered and uploaded to Google Forms to ensure consistency in data management and analysis.

### Measures and Instruments

Conceptual understanding will be measured using a 5-item adapted scale from the Conceptual Understanding Inventory (Cronbach α=0.82) [26]. Critical thinking confidence will be assessed via the critical thinking disposition subscale of the California Critical Thinking Disposition Inventory (Cronbach α=0.89) [27]. AI dependency will be measured using a newly developed 6-item scale based on cognitive offloading literature [28], with pilot data indicating a Cronbach α value of 0.78. AI literacy will be assessed using the Digital AI Literacy Scale (Cronbach α=0.85) [29]. All scales use 5-point Likert responses; composite scores will be calculated as means. New items underwent expert review (n=3) and cognitive interviewing (n=10) during pilot-testing.

### Data Analysis

#### Qualitative Data

In line with the exploratory-descriptive qualitative design of this study, data analysis will be performed iteratively and inductively alongside data collection, as illustrated in Table 1. As interviews are transcribed, the researcher will repeatedly and carefully read each transcript to immerse themselves in participants’ descriptions and develop an overall understanding of the data. This initial familiarization will support an open exploration of participants’ experiences, allowing meanings to emerge directly from the data without imposing predetermined categories.

The analysis will start with a detailed review of one transcript, during which the researcher will reflect on what the participant describes and consider the deeper meanings in the story. Interpretive notes and initial thoughts will be written in the margins to capture emerging insights. This process will be systematically repeated for all transcripts, enabling ongoing comparison and refinement of understanding as new data are gathered.

As the analysis advances, recurring ideas and descriptive patterns will be identified and compiled into a list of topics. Similar topics will be grouped and gradually refined into themes and subthemes that accurately represent participants’ accounts. The researcher will then revisit the transcripts and assign short codes to relevant segments of text, ensuring that the analysis remains closely connected to participants’ words and descriptions. Throughout the process, the researcher will stay attentive to the emergence of new codes or themes, allowing the analytical framework to adapt as understanding deepens. Guided by the qualitative data analysis method by Tesch [25], this exploratory-descriptive approach will focus on detailed, contextualized descriptions of participants’ experiences, ensuring that the findings accurately reflect the phenomena under investigation.

#### Reliability and Validity

A pilot study will be conducted to evaluate the feasibility of the survey questionnaire, which will be developed as part of the study’s quantitative data collection tools. Sample size was determined a priori using G*Power (version 3.1). For detecting a small to medium effect (*f*=0.15) in multiple regression with 8 predictors, α of .05, and power of 0.80, a minimum of 228 participants is required. Starting from the base requirement of 228 participants, we applied a design effect of 1.3 to account for departmental clustering (228 × 1.3 = 296), then inflated for an anticipated 30% nonresponse rate (296/0.70 ≈ 423); the final target of 384 reflects a pragmatic balance between statistical ideals and feasible recruitment within the single-institution context. For pretest-posttest intervention analyses, a paired *t* test with Cohen *d*=0.35 required 199 participants; inflating for attrition yields a target of 285 for the intervention subsample. The internal consistency of multi-item scales will be assessed using the Cronbach α (threshold of ≥0.70). Where scales are administered at 2 time points, test-retest reliability will be evaluated using intraclass correlation coefficients. Logistic regression will be reserved for modeling binary outcomes (eg, high vs low AI dependency) as a function of predictor variables, not for reliability assessment.

#### Trustworthiness

The study’s trustworthiness will be established through multiple methods to ensure the rigor and robustness of the qualitative data collected. In line with a social constructivist approach, the researcher will aim to triangulate data to capture multiple perspectives. Data source triangulation will involve collecting data from different student groups across the 2 faculties (Applied Sciences and Health Sciences) and the 3 departments (Mathematics, Somatology, and Biomedical and Clinical Technology) using multiple qualitative data collection methods. Data triangulation will be achieved through focus groups, semistructured interviews, and document reviews. Additional strategies will include peer debriefing and authentic member checking. To facilitate peer debriefing, the researcher will review codes and themes with the study’s coinvestigators to promote reflexivity and ensure that all data are considered. Authentic member checking will involve summarizing key points at the end of focus groups and interviews and asking participants to verify the accuracy of the data. These strategies will help establish the credibility of the data. Data reliability will be maintained through an audit trail, with the researcher keeping field notes from participant interviews.

#### Statistical Analyses and Rationale

In consultation with a statistician, the quantitative analysis (Table 1) will begin with descriptive statistics, including frequencies and percentages for categorical variables (such as AI tool type, academic purpose, discipline, and year level) and means with SDs for composite Likert-type scales. These summaries characterize the sample and establish baseline patterns of AI use before conducting inferential testing. Group associations among categorical variables (eg, purpose × year level; tool type × discipline) will be examined using cross-tabulations with chi-square tests of association, which are suitable for testing independence in contingency tables. When expected counts are sparse, the Fisher exact test will be applied. Internal consistency of multi-item scales (eg, perceptions of impact, risk awareness, ethical awareness, and AI literacy) will be assessed using the Cronbach α to justify the creation of reliable composite scores prior to conducting correlational or group analyses. Relationships among continuous or ordinal composite scores (such as perception, critical thinking confidence, AI dependency indicators, verification behaviors, literacy and ethics scores, and AI use frequency) will be analyzed using the Pearson *r* when normality and interval-level assumptions are approximately met; otherwise, the Spearman ρ will be used because it is robust to nonnormality and ordinal measurement. For the AI literacy intervention, within-participant pretest-posttest change will be assessed using a paired *t* test (suitable for mean differences on approximately normally distributed change scores); if normality is not met, the nonparametric Wilcoxon signed-rank test will be used.

All hypothesis tests will be 2-tailed at an α value of .05, with effect sizes reported (eg, Cramér *V* for chi-square tests, *r* for Spearman ρ, and Cohen *d* for paired comparisons) and 95% CIs to indicate practical significance. Assumptions will be checked (eg, expected cell counts for chi-square tests and Shapiro-Wilk for change scores in paired tests). When multiple families of tests are performed (eg, several cross-tabulations across departments), a Holm-Bonferroni adjustment will be considered to control the familywise type I error rate. For Likert items, inference will be conducted on validated composites (rather than individual items) to better approximate interval scaling; item-level results, if explored, will be reported descriptively.

### Statistical Limitations

As key constructs rely on self-report, findings may be subject to social desirability and recall bias. To mitigate this, survey items use neutral wording, anonymity is emphasized, and triangulation with qualitative data allows for cross-validation of reported behaviors.

### Ethical Considerations

The fundamental principles of the Declaration of Helsinki will be observed throughout this research. All participants will sign an informed consent form after being informed about the study procedures indicating their willingness to participate (Multimedia Appendix 2). The consent form details the study's benefits and risks, which include respect, self-determination, informed decision-making, and request permission to record audio during interviews for data collection. Participants will also be reminded of their right to withdraw at any time during the study. Beneficence will be maintained by supporting participants’ well-being throughout the research.

Student confidentiality will be protected through deidentification of all survey and interview data at the point of collection; identifiers will be stored separately on a password-protected server accessible only to the principal investigator. To avoid coercion, recruitment materials will emphasize voluntary participation and clarify that nonparticipation carries no academic penalty. Sensitive disclosures regarding AI misuse will be handled by providing participants with institutional academic integrity resources without recording identifiable details. Audio recordings will be transcribed verbatim, anonymized, and stored on encrypted university servers; raw audio files will be deleted after transcription verification. All data will be retained for 5 years per DUT policy before secure destruction. Gatekeeper permission has been obtained through the DUT Research and Postgraduate Support Directorate (Multimedia Appendix 3). The study has received full ethics approval from DUT’s Institutional Research Ethics Committee with ethical clearance number IN-IREC 082/25 (Multimedia Appendix 4).

## Results

The pilot study was funded by the Department of Higher Education and Training on January 1, 2025, and was conducted between August 2025 and November 2025, with preliminary results available at the end of that period. Data collection is ongoing and is expected to conclude by the end of November 2026, after which the data will be analyzed in 2027 and the findings will be published in a separate results paper.

The anticipated findings of this study are expected to reveal significant patterns in the use of AI tools among undergraduate students across the participating departments. Drawing on preliminary evidence from a pilot study conducted within the DUT Department of Mathematics, it is anticipated that students will demonstrate a substantial reliance on AI-driven platforms, particularly for problem-solving and assessment-related tasks. These early observations suggest a growing dependence on AI, which may inadvertently encourage surface-level engagement with learning material, limit independent reasoning, and constrain the development of critical thinking skills. Building on these initial insights, the full study is expected to provide a more comprehensive and contextually grounded understanding of students’ AI use practices, thereby informing the development of responsive academic guidelines and potential curriculum interventions tailored to the institution’s educational context.

The project will be further piloted and implemented in collaboration with the Center for Excellence in Learning and Teaching to help develop a short course on responsible and pedagogically sound use of AI. The expected outcomes of this phase include evidence of increased AI literacy, heightened ethical awareness, and more reflective use of AI among participating students. Overall, the results are expected to inform institutional practices by providing evidence-based recommendations for AI integration, curriculum support, and professional development initiatives that align with DUT’s Envision 2030 strategy and the broader UoT mandate.

## Discussion

### Anticipated Findings

The discussion is expected to build on emerging evidence from the pilot findings, which indicate that students are already navigating both the benefits and limitations of AI use. While AI is recognized as a valuable tool for enhancing access to learning resources and providing personalized academic support, concerns have been raised regarding ethical use, academic integrity, and appropriate boundaries, particularly within applied and health-related disciplines. These preliminary insights, together with the anticipated broader dataset [23,24], suggest that aligning with the DUT goal of producing adaptable, technologically proficient, and ethically grounded graduates requires targeted AI literacy interventions that strengthen critical understanding, promote responsible use, and foster reflective engagement with AI technologies.

Within this context, both the Faculty of Applied Sciences and the Faculty of Health Sciences at DUT operate under the institutional vision articulated in Envision 2030 [22] and under broader higher education imperatives shaped by national and international standards [23,24]. As a UoT, DUT is uniquely positioned to emphasize applied knowledge, technological innovation, and work-integrated learning. The anticipated findings are therefore expected to highlight gaps in students’ critical and ethically grounded engagement with AI tools, reinforcing the need for structured and context-responsive AI integration strategies across practice-based disciplines.

Drawing on current literature on AI in higher education [30,31], the study is expected to demonstrate widespread yet largely informal and unregulated use of AI among undergraduate students, particularly for efficiency-driven academic tasks such as information retrieval, summarization, and assessment preparation. In a UoT setting, where learning is closely linked to professional competence and practical application [32,33], such patterns may raise concerns about superficial engagement with disciplinary knowledge, uneven conceptual development, and a potential decline in independent problem-solving abilities. Variations across disciplines and levels of study are also anticipated, with senior students likely to show greater confidence in using AI and a higher propensity to rely on it, especially in applied learning contexts.

Overall, the discussion is intended to position AI not as a substitute for applied learning or professional judgment but as a complementary tool that requires intentional pedagogical framing. When effectively guided by institutional policies and curriculum design, AI can support the development of practice-ready graduates while maintaining academic integrity and disciplinary standards. In doing so, the study contributes to the ongoing discourse on how UoTs can strategically integrate AI to enhance applied pedagogy, uphold professional accountability, and advance institutional goals within an increasingly digital higher education landscape.

### Strengths, Limitations, and Future Directions

A key strength of this study lies in its mixed methods design, which enables a comprehensive examination of undergraduate students’ use and perceptions of AI by integrating quantitative trends with qualitative insights. The inclusion of multiple departments within the Faculties of Applied Sciences and Health Sciences strengthens the study’s relevance to the UoT context, where applied learning, technological engagement, and graduate readiness are central. In addition, the study’s alignment with DUT’s Envision 2030 strategy enhances its institutional relevance and its potential to inform curriculum development, AI literacy initiatives, and policy-aligned practice [22].

Notwithstanding these strengths, certain limitations are acknowledged. The study is situated within a single UoT, which may limit the generalizability of the findings to other institutional contexts. Furthermore, reliance on self-reported data may be subject to response and social desirability bias, particularly when students report AI use and ethical practices. The cross-sectional nature of the study also limits conclusions regarding causality or the longer-term impacts of AI use on learning.

These limitations point to several directions for future research. Subsequent studies could adopt comparative or multi-institutional designs to explore similarities and differences in AI use across UoTs and traditional universities. Longitudinal or intervention-based research would further support examination of how AI literacy initiatives influence students’ learning practices, ethical awareness, and professional competence over time. Future research could also incorporate learning artifacts or analytics data to complement self-reported measures and deepen understanding of AI’s role in applied and practice-based education.

### Conclusions

This study aims to contribute to the growing body of knowledge on the use of AI in undergraduate education by exploring students’ practices, perceptions, and ethical engagement within a UoT setting. By framing the research within applied and health-related fields and aligning it with DUT’s Envision 2030 strategy, the study is well positioned to yield relevant insights that support responsible and effective AI integration in teaching. The results are expected to inform evidence-based decisions on AI literacy, curriculum development, and institutional policies while also advancing academic understanding of how AI can enhance learning without compromising academic integrity or professional skills.

## Supplementary material

10.2196/92990 Multimedia Appendix 1 Study questionnaire.

10.2196/92990 Multimedia Appendix 2 Gatekeeper permission.

10.2196/92990 Multimedia Appendix 3 Ethics clearance.

10.2196/92990 Multimedia Appendix 4 Letter of information and informed consent.

## Abbreviations

BHSc: Bachelor of Health Sciences
DUT: Durban University of Technology
LMS: learning management system
UoT: university of technology
