# Supplementary material for: Analysis of Student Engagement With AI in Undergraduate Lectures: Protocol for an Implementation Study
Source: JMIR Res Protoc. 2026 Oct 21;15:e92990. doi: 10.2196/92990 (PMC13686339; doi:10.2196/92990)
Supplement: Supplementary file 2 [file resprot-v15-e92990-s002.docx]

26 November 2025 Dr M Ntuli

Faculty of Applied Sciences Durban University of Technology

Dear Dr Ntuli

**Directorate for Research and Postgraduate Support**

3rd Floor, Open House, Steve Biko Campus Durban University of Technology

P O Box 1334, Durban, 4000

**T** +27 373 2577

**E** [*Gatekeeper@dut.ac.za*](mailto:Gatekeeper@dut.ac.za)

**W** https://[www.dut.ac.za/research-ps/](http://www.dut.ac.za/research-ps/)

PERMISSION TO CONDUCT RESEARCH AT THE DURBAN UNIVERSITY OF TECHNOLOGY

Your email correspondence in respect of the above refers. I am pleased to inform you that the Institutional Research and Innovation Committee (IRIC) has granted Gatekeeper Permission for you to conduct your research “Analysis of Student Engagement with AI in Undergraduate Lectures” at the Durban University of Technology. **Kindly note that this letter must be issued to the IREC for approval before you commence data collection**.

The Durban University of Technology may impose any other condition it deems appropriate in the circumstances having regard to nature and extent of access to and use of information requested.

Upon completion of your research project, you are requested to share the summary of your key research findings.

Yours sincerely

Dr N H Mthombeni Director (Interim)

Research and Postgraduate Support
