# Supplementary material for: Analysis of Student Engagement With AI in Undergraduate Lectures: Protocol for an Implementation Study
Source: JMIR Res Protoc. 2026 Oct 21;15:e92990. doi: 10.2196/92990 (PMC13686339; doi:10.2196/92990)
Supplement: Supplementary file 3 [file resprot-v15-e92990-s003.docx]

2 December 2025 Dr M Ntuli

Faculty of Applied Sciences Durban University of Technology

Dear Dr Ntuli

Institutional Research Ethics Committee Research and Postgraduate Support Directorate Ground Floor Block B

ML Sultan Campus

Durban University of Technology

P O Box 1334, Durban, South Africa, 4001 Tel: 031 373 6755

Email: [Irec@dut.ac.za](mailto:Irec@dut.ac.za) <http://www.dut.ac.za/research/institutional_research_ethics>

[www.dut.ac.za](http://www.dut.ac.za/)

# Analysis of Student Engagement with AI in Undergraduate Lectures Ethical Clearance number: IN- IREC 082/25

The DUT-Institutional Research Ethics Committee acknowledges receipt of your gatekeeper permission letter.

Please note that FULL APPROVAL is granted to your research proposal. You may proceed with data collection.

Any adverse events [serious or minor] which occur in connection with this study and/or which may alter its ethical consideration must be reported to the DUT-IREC according to the DUT-IREC Standard Operating Procedures (SOP’s).

Please note that any deviations from the approved proposal require the approval of the DUT-IREC as outlined in the DUT-IREC SOP’s.

# It is compulsory for a student or researcher to apply for recertification on an annual basis. The failure to do so will result in withdrawal of ethics clearance. It is the responsibility of the researcher and the supervisor to apply for recertification.

**Please note that you are required to submit a Notification of Completion of Study form together with an abstract to the DUT-IREC office on completion of your study.**

Yours Sincerely

Professor P Mashau Chairperson: DUT-IREC
