# Supplementary material for: Analysis of Student Engagement With AI in Undergraduate Lectures: Protocol for an Implementation Study
Source: JMIR Res Protoc. 2026 Oct 21;15:e92990. doi: 10.2196/92990 (PMC13686339; doi:10.2196/92990)
Supplement: Supplementary file 4 [file resprot-v15-e92990-s004.docx]

**LETTER OF INFORMATION**

**Title of the Research Study:** Analysis of Student Engagement with AI in Undergraduate Lectures

**Principal Investigator/s/researcher: Dr.** Mthobisi Ntuli (PhD in Applied Mathematics)

**Co-Investigator/s/supervisor/s:** Dr Shamanie Govender, Dr. Godfrey Mazibuko, and Dr Khosi Mpofana

# Brief Introduction and Purpose of the Study:

My name is Mthobisi Ntuli, and my co investigators, are conducting the above-mentioned study through the **Department of Mathematics, Biomed and Clinical Technology AND Somatology** at the **Durban University of Technology.** I would like to invite you to participate in a study that explores how students use Artificial Intelligence (AI) tools such as ChatGPT, Microsoft Copilot, and Wolfram Alpha during lectures.

# What is Research

Research is a systematic process of inquiry aimed at generating new knowledge. In this study, we aim to understand how AI affects students thinking, problem-solving, and student engagement.

You are welcome to ask any questions about the study. You may also discuss your participation with family or friends before deciding. A copy of this letter is provided for you to take home.

# Outline of the Procedures:

You will be asked to complete a short questionnaire and may be invited to participate in an interview or classroom observation. The study will explore your use of AI tools in learning, your perceptions, and your reasoning processes. Participation is voluntary and will take approximately 30–45 minutes. Around 50–200 students will be involved. No treatment or randomisation is involved.

**Risks or Discomforts to the Participant:** There are no known risks or physical discomforts associated with this study. All efforts will be made to ensure your comfort and privacy.

# Explain to the participant the reasons he/she may be withdraw from the Study:

You may withdraw from the study at any time without penalty. The researcher may also withdraw you if necessary (e.g., illness or non-compliance). If you choose to withdraw, your data will not be used.

# Benefits:

You may benefit directly by gaining insights into AI tools and improving your learning. Indirectly, your participation will help improve teaching practices and curriculum design.

# Remuneration:

There is no monetary compensation for participation.

# Costs of the Study:

There are no costs to you for participating in this study.

**Confidentiality:** Full confidentiality and anonymity will be ensured in all transcripts and data collections by the use of a *pseudonym* (i.e. fictitious name) for each participant. Thus, the identities of all interviewees will be kept strictly confidential. In addition, all data collected will be securely stored until completion of the study, after which it will be transferred to locked storage at DUT for a period of five years, and then disposed of. Only The researcher will have access.

# Results:

The results will be shared through academic publications and presentations. If significant findings arise during the study, they will be communicated to you.

# Research-related Injury:

There are no physical procedures involved, so injury is unlikely. No compensation is applicable.

# Storage of all electronic and hard copies including tape recordings:

Access will be restricted to the research team. After this period, data will be securely destroyed.

# Persons to contact in the Event of Any Problems or Queries:

Researcher: Mthobisi Ntuli – [mthobisin2@dut.ac.za](mailto:mthobisin2@dut.ac.za)

DUT-Institutional Research Ethics Administrator on 031 373 2375. Complaints can be reported to the Acting Director: Research and Postgraduate Support on [researchdirector@dut.ac.za](mailto:researchdirector@dut.ac.za)

# General:

**(This section must be deleted before attaching document to your PG 2a)**

A copy of the information letter should be issued to participants. The information letter and consent form must be translated and provided in the primary spoken language of the research population e.g. isiZulu.

# CONSENT

**Full Title of the Study:** Analysis of Student Engagement with AI in Undergraduate Lectures

**Names of Researcher:** Dr. Mthobisi Ntuli

# Statement of Agreement to Participate in the Research Study:

- I hereby confirm that I have been informed by the researcher, Dr. Ntuli, Dr Shamanie Govender, Dr. Godfrey Mazibuko, and Dr Khosi Mpofana (Strike through your lecturer) about the nature, conduct, benefits and risks of this study - Research Ethics Clearance

Number:	_,

- I have also received, read and understood the above written information (Participant Letter of Information) regarding the study.
- I am aware that the results of the study, including personal details regarding my sex, age, date of birth, initials and diagnosis will be anonymously processed into a study report.
- In view of the requirements of research, I agree that the data collected during this study can be processed in a computerised system by the researcher.
- I may, at any stage, without prejudice, withdraw my consent and participation in the study.
- I have had sufficient opportunity to ask questions and (of my own free will) declare myself prepared to participate in the study.
- I understand that significant new findings developed during the course of this research which may relate to my participation will be made available to me.

# Full Name of Participant Date Time Signature / Right Thumbprint

I,	(name of researcher) herewith confirm that the above participant has been fully informed about the nature, conduct and risks of the above study.

**Full Name of Researcher	Date	Signature**

**Full Name of Witness (If applicable)	Date	Signature**

**Full Name of Legal Guardian (If applicable) Date	Signature**

## Please note the following:

Research details must be provided in a clear, simple and culturally appropriate manner and prospective participants should be helped to arrive at an informed decision by use of appropriate language (grade 10 level- use Flesch Reading Ease Scores on Microsoft Word), selecting of a non- threatening environment for interaction and the availability of peer counselling (Department of Health, 2004).

If the potential participant is unable to read/illiterate, then a right thumb print is required and an impartial witness, who is literate and knows the participant e.g. parent, sibling, friend, pastor, etc. should verify in writing, duly signed that informed verbal consent was obtained (Department of Health, 2004).

If anyone makes a mistake completing this document e.g. a wrong date or spelling mistake, a new document has to be completed. The incomplete original document has to be kept in the participant’s file and not thrown away, and copies thereof must be issued to the participant.

## References:

Department of Health: 2004. *Ethics in Health Research: Principles, Structures and Processes* <http://www.doh.gov.za/docs/factsheets/guidelines/ethnics/>

Department of Health. 2006. *South African Good Clinical Practice Guidelines*. 2nd Ed. Available at: <http://www.nhrec.org.za/?page_id=14>

# NOTE: Information in brackets in the Letter of Information is to be used as a guide in completing the Letter of Information. This information as well as the general section at the end of the Letter of Information and Consent document must be deleted before attaching the document to the PG 2a.

9 June 2022
