# Supplementary material for: Analysis of Student Engagement With AI in Undergraduate Lectures: Protocol for an Implementation Study
Source: JMIR Res Protoc. 2026 Oct 21;15:e92990. doi: 10.2196/92990 (PMC13686339; doi:10.2196/92990)
Supplement: Supplementary file 5 [file resprot-v15-e92990-s001.docx]

Appendix 1
